# Measuring Exercise-Induced Secreted Protein Acidic and Rich in Cysteine Expression as a Molecular Tool to Optimize Personalized Medicine

**DOI:** 10.3390/genes12111832

**Published:** 2021-11-20

**Authors:** Abdelaziz Ghanemi, Mayumi Yoshioka, Jonny St-Amand

**Affiliations:** 1 Functional Genomics Laboratory, Endocrinology and Nephrology Axis, CHU de Québec-Université Laval Research Center, Québec, QC G1V 4G2, Canada; abdelaziz.ghanemi@crchudequebec.ulaval.ca (A.G.); mayumi.yoshioka@crchudequebec.ulaval.ca (M.Y.); 2 Department of Molecular Medicine, Faculty of Medicine, Laval University, Québec, QC G1V 0A6, Canada

**Keywords:** exercise, secreted protein acidic and rich in cysteine, expression, medicine

## Abstract

The numerous exercise benefits for health as well as applications for diseases has lead to exercise being prescribed in many pathological conditions. Secreted protein acidic and rich in cysteine (SPARC) gene expression is stimulated by exercise and SPARC has been suggested as a molecular mediator of exercise. Therefore, we suggest using this property for personalized medicine. This can be achieved by prescribing the exercise with a pattern (duration, intensity, etc.) that corresponds to the optimum *SPARC/Sparc* expression. We expect this approach to optimize the exercise therapy in both the preventive and curative contexts. In the research field, measuring exercise -dependent expression of *Sparc* would represent a molecular tool to further optimize the selection of exercise animal models as well.

With the development of non-pharmacological and non-surgical approaches in therapeutics, the medical applications of exercise are gaining increasing importance. Indeed, beyond being a habit for numerous individuals with positive impacts on mood [1,2], exercise represents a therapeutic option for a variety of diseases and health conditions. It has been used within medical protocols either as a therapy or as an adjuvant to treat, prevent or improve diseases and health problems in which effects including controlling energy balance or enhancing biological properties are therapeutic targets such as cardiovascular diseases [3,4], obesity [5,6,7,8], low back pain [9], metabolic disorders [10], chronic kidney disease [11], regeneration [12], cancer [13], diabetes [14], immunity and infections [15,16,17]. Such exercise applications find their origin in the very numerous benefits that exercise has on health. This includes lowering blood pressure [18], bone osteogenesis stimulation [19], reducing cachexia [11] and anti-inflammatory effects [20]. Mental health (anxiety, stress and depression), sports psychiatry [21,22,23], and improved sleep quality [24,25] are also in this list. These medical benefits were considered as “granted” for humans who lived before the current industrial area because they had a healthier lifestyle that included sufficient physical activity. Thus, it significantly contributed to positive public health. However, in the last decades, the development of technologies has made life easier and humans need less effort to achieve what required huge effort previously. This situation has lead to a sedentary lifestyle and less active societies, which has contributed to the increase of various humans diseases. As an attempt to correct this negative consequence of modernity, health professionals are recommending physical activity for diverse population categories. 

Regarding the molecular mechanism linking exercise and the exercise-induced effects, exercise benefits have been suggested to be mediated through a variety of factors, mainly the muscle-secreted myokines [26] that are produced by skeletal muscles and increase in response to exercise [27]. Such an exercise-induced pattern of secretion suggests that these myokines would govern the molecular pathways underlying the phenotypic changes resulting from exercise in different organs and tissues leading to the known health benefits of the physical activity. May be the most interesting one is secreted protein acidic and rich in cysteine (SPARC), an exercise-responsive myokine [28] in both humans and mice [29]. Indeed, using powerful functional genomics that represent a strong strategy to study the dynamic expression of genes [30,31], *SPARC* has been characterized as an exercise-induced gene [32]. Initially, the serial analysis of gene expression revealed that the cycle ergometer training increased the *SPARC* expression in muscles following endurance training [32]. Moreover, Aoi el al. showed that a single bout of exercise increased SPARC expression in the muscle and also in the plasma [29] and such a plasma exercise-induced increase becomes more important following training [29]. 

Following that, in vitro studies have been performed on C2C12 muscle cells to further explore SPARC-exercise mechanistic links. Electrical pulse stimulation (EPS), considered as the in vitro model of exercise [33,34,35,36], applied on C2C12 cells also induced *Sparc* expression [37]. The same in vitro studies showed that *Sparc* modulates mitochondrial functions [37] and that adding SPARC both increased myoblasts differentiation and mitochondrial proteins in C2C12 cells [38]. Importantly, a recent in vivo study on trained *Sparc* knock-out mice suggested that exercise-induced muscle phenotype changes, including metabolism, strength and development, are SPARC-dependent [39]. Together, these data highlight SPARC as a key mediator of the exercise-induced benefits. Furthermore, the roles and functions in which SPARC has been implicated or suggested to be involved correlate with exercise effects. For instance, beyond its known implications, mainly in tissue repair [40], SPARC has been suggested to be involved in metabolic changes [28,41,42,43,44], bone formation [45], regeneration [46,47,48], anticancer effects [29,49], anti-inflammatory paths [50], and regulating muscle mass and function [51], all of which have also been shown to improve with exercise; which further supports the existence of molecular links between SPARC functions and exercise effects [28,52]. These cellular and molecular properties may represent the rationale why *SPARC*/*Sparc* functions as an exercise-responsive gene and why SPARC is induced by exercise. Indeed, since exercise may promote health and enhance systemic health via various cellular responses (e.g., metabolic change, bone, regeneration, anti-cancer, anti-inflammatory and regulation of muscle mass and function) that have been shown to implicate SPARC, SPARC comes out as a molecular mediator secreted following exercise to enhance and stimulate biological properties and endogenous processes toward a healthy homeostatic phenotype.

Therefore, since exercise effects are mediated via SPARC, the optimum exercise would be the one that induces SPARC/*SPARC/Sparc* expression the most. Thus, we suggest—for the first time to our knowledge—applying such a concept for personalized medicine. The process would be to challenge individuals with a variety of exercise patterns and programs that are different in terms of type of exercise type, the used device, the speed, duration, time (morning, night, etc.) and even the addition of other factors such as the temperature and incline setting (treadmill) for instance. Following the exercise, we proceed to a muscle biopsy, a common procedure [53,54], to measure the expression of *SPARC/Sparc*. Based on the results, the optimum exercise conditions (time, speed, environment, etc.) would be determined as those corresponding to the optimum *SPARC/Sparc* expression. Future studies, would allow one to make further links not only between exercise and *SPARC/Sparc* gene expression but also between the exercise and the protein SPARC expression or its serum levels that increase following exercise [29,55], thus adding the protein expression and the serum concentrations of SPARC as novel exercise-efficacy evaluation tools. Such tools would allow one to estimate the benefits that an exercise (depending on its patterns) would induce and open the door to a variety of potential applications (Figure 1). Measuring exercise-induced SPARC/*SPARC*/*Sparc* can contribute to answering the questions discussed in diverse studies in terms of exercise “dose” [14,56,57,58]. Indeed, a possible correlation between the exercise intensity and SPARC serum level has been shown [59], which supports such SPARC-dependent evaluation of exercise effects.

For clinical perspectives, which still require deeper investigations, the main application would be to determine the optimum parameters of the exercise to prescribe for patients suffering from diseases and health problems for which exercise represents a therapy. Of course, the tested exercise intensity, duration, strength, etc. would depend on each patient based on the physiological and biochemical parameters that limit the exercise ability such as oxygen saturation, lung capacity, heart status, glycemia and physical disabilities. Indeed, going beyond those physiological limits will not only be harmful but could also have no exercise-induced benefits, as suggested by the fact that supramaximal exercise had no effect on SPARC levels [60]. Such a need to set a limit could be achived by measuring SPARC via the evaluation of exercise-dependant SPARC expression as well. 

Another application would be the optimization of animal models of exercise to better develop exercise science and exercise-related research towards an optimized application of exercise to treat patients, as a prevention for healthy individuals or to optimize training efficacy and outcome for athletes. Furthermore, the same *SPARC/Sparc* expression as a measure of exercise efficacy principle can be used not only to optimize the exercise pattern but also to compare different groups based on age, sex, diet, genetic polymorphism and species (animals). In this context, the in vitro models of exercise (electric pulse stimulation) would also provide additional data at the molecular and subcellular levels.

The importance of such new tools comes from the fact that exercise represents a “panacea” for limitless health problems. We believe that this suggested approach of measuring of SPARC/*SPARC/Sparc* expression/level in response to different exercise patterns could optimize exercise science and provide molecular evaluation tools to significantly improve public health via personalized medicine. One of the main applications would be to manage obesity and metabolic disorders. This concept would also be of a specific application for the older population that have many potential benefits from exercise but that need to be optimized in terms of intensity, type and duration for healthy ageing [61,62,63]. 

The evidence we have provided builds up a puzzle that suggests SPARC as a selective biological marker that reflects the physiological responsiveness to exercise, not only through muscle-related patterns [64,65,66,67] but also metabolism [68], adiposity [69], and other effects, and thus the quality and the level of the induced benefits we will see following that exercise. However, further studies are still required to confirm and quantify the exact correlation between SPARC/*SPARC* expression and the various factors that define the exercise amount, mainly the intensity and the duration. These studies should focus on numerical and quantitative correlation similar to what we have for biomarkers used in clinical practice or biomedical research [70,71,72,73,74,75,76,77,78,79,80,81,82,83,84,85,86,87] including SPARC itself, which was also suggested as a physiological and pathological biomarker [88]. Personalized medicine [89,90,91,92,93] and precision medicine [94,95,96,97,98,99,100,101] are growing areas in respect to exercise [102,103,104,105,106,107], which further highlights the potential of measuring SPARC/*SPARC*/*Sparc* expression/level in optimizing and developing medical practice.

## Figures and Tables

**Figure 1:**
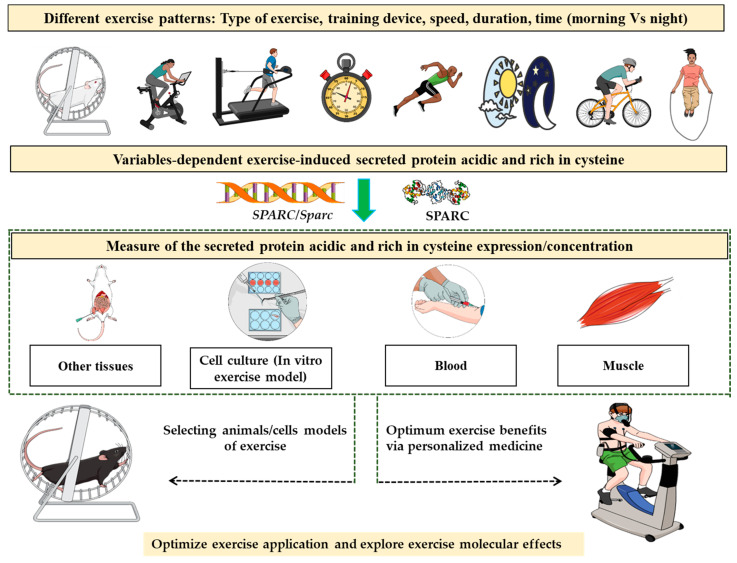
Measuring secreted protein acidic and rich in cysteine expression/concentration in biological samples following different patterns of exercise training would reflect the biological “responsiveness” to the physical activity and would predict the intensity of the benefits that exercise-induced changes will have. Such a property could be explored for instance to optimize the prescribed physical activity towards a personalized medicine approach and also select animal/cell models of exercise.

## Data Availability

Not applicable.
